# Characterization of Cecal Smooth Muscle Contraction in Laying Hens

**DOI:** 10.3390/vetsci8060091

**Published:** 2021-05-26

**Authors:** Katrin Röhm, Martin Diener, Korinna Huber, Jana Seifert

**Affiliations:** 1 Institute of Animal Science, University of Hohenheim, 70593 Stuttgart, Germany; katrin.roehm@uni-hohenheim.de (K.R.); korinna.huber@uni-hohenheim.de (K.H.); 2 Institute of Veterinary Physiology and Biochemistry, Justus-Liebig University, 35392 Gießen, Germany; martin.diener@vetmed.uni-giessen.de

**Keywords:** *Gallus gallus*, laying hen, smooth muscle, contraction, ceca

## Abstract

The ceca play an important role in the physiology of the gastrointestinal tract in chickens. Nevertheless, there is a gap of knowledge regarding the functionality of the ceca in poultry, especially with respect to physiological cecal smooth muscle contraction. The aim of the current study is the ex vivo characterization of cecal smooth muscle contraction in laying hens. Muscle strips of circular cecal smooth muscle from eleven hens are prepared to investigate their contraction ex vivo. Contraction is detected using an isometric force transducer, determining its frequency, height and intensity. Spontaneous contraction of the chicken cecal smooth muscle and the influence of buffers (calcium-free buffer and potassium-enriched buffer) and drugs (carbachol, nitroprusside, isoprenaline and Verapamil) affecting smooth muscle contraction at different levels are characterized. A decrease in smooth muscle contraction is observed when a calcium-free buffer is used. Carbachol causes an increase in smooth muscle contraction, whereas atropine inhibits contraction. Nitroprusside, isoprenaline and Verapamil result in a depression of smooth muscle contraction. In conclusion, the present results confirm a similar contraction behavior of cecal smooth muscles in laying hens as shown previously in other species.

## 1. Introduction

The pairs of ceca in the avian intestine play a dominant role in digestion as they are the major location of fermentation [1] with the highest bacterial diversity [2,3]. Furthermore, they are responsible for water and electrolyte absorption [4,5,6,7] and are involved in the nitrogen metabolism of the chicken [8]. Rectal antiperistaltic waves enable the transport of urine in the ceca [9], which is a source of water, electrolytes and nitrogen for the synthesis of microbial amino acids [10].

Intestinal peristalsis is further essential to mix the digesta and to move it along the intestinal tube [11,12]. It plays an important role in microbial cell density and the elimination of pathogens [13,14]. It was shown that the systemic inhibition of intestinal contraction by morphine leads to bacterial overgrowth in the duodenum and jejunum [14].

Experiments with turkeys indicated that contractions of low frequency and amplitude to mix the digesta and larger contractions for emptying the ceca are present [15,16]. In broiler chickens, spontaneous cecal smooth muscle contraction differed with the extent of cecal distension [17,18]. Furthermore, the polarity of the peristaltic reflex in birds is different to mammals, as it expands in two-way directions, oral and aboral, and not in a one-way, aboral direction [17]. 

Any kind of peristaltic motility in the intestinal tract depends on the contraction and relaxation of the intestinal smooth muscle. The tunica muscularis of birds, which is the outer layer of the intestinal wall, consists of four muscle layers: inner circular and longitudinal and outer circular and longitudinal muscle [19]. Some of the muscle cells are connected to each other by gap junctions, building a functional syncytium. This is an autonomous system as it also includes pacemaker cells, along with interstitial cells of Cajal, which generate electrical activity (slow waves) [11,12]. Contractions are triggered by the enteric nervous system as it modulates the membrane potential by inhibitory and exhibitory neurotransmitters [12], as well as by the extrinsic autonomous nervous system, including fibers of the sympathetic and parasympathetic nervous system [11]. Furthermore, smooth muscle contraction in the gut is influenced by hormones [12].

The contraction of smooth muscles is calcium-dependent. Excitatory signals cause the influx of Ca^2+^ from the extracellular space via calcium channels and the release of intracellular Ca^2+^. Ca^2+^ is bound to calmodulin, a calcium-binding receptor protein, leading to the activation of the myosin light chain kinase (MLCK) by the Ca-calmodulin complex. MLCK phosphorylates the regulatory myosin light chain (MLC), which stimulates myosin ATPase and thus facilitates cross-bridge cycling and the contraction of smooth muscle. The dephosphorylation of MLC leads to a reduction of cross-bridge cycling and termination of the muscle contraction [20].

Smooth muscle contraction can be measured ex vivo. The muscle is fixed in an organ bath and the isometric contraction is recorded using a force transducer. This kind of measurement was used before to investigate the physiological functions of the smooth muscle in animals and humans [21,22,23] and the impact of different drugs on smooth muscle contraction [24,25,26]. It was also successfully used to study the in vitro contractile activity of intact cecal segments from Ross 308 broilers [18].

Shifts in the ion balance and different substances of the enteric and extrinsic autonomous nervous systems can influence smooth muscle contraction, leading to physiologic and pathophysiologic changes to the contraction in vivo. These changes can also be evoked in vitro.

Despite the major role of the ceca for digestion processes in chickens, there is still a lack of knowledge about the physiology of contraction in the respective smooth muscle. The aim of this study was the ex vivo characterization of physiological smooth muscle contraction in the ceca, and the muscular response to different electrolytes and drugs in laying hens.

## 2. Materials and Methods

### 2.1. Animals and Feeding

The protocol for animal treatment was approved and its execution supervised by the animal welfare officer of the University of Hohenheim, Germany (184/19 AP). Eleven laying hens (Lohmann LSL-Classic (LSL; N = 4) and Lohmann brown classic (LB; N = 7), Fa. Lohmann Tierzucht, Cuxhaven) were housed in the barn of the Institute of Animal Science at the University of Hohenheim, Germany, in groups under free-range conditions. They were fed ad libitum with a commercial diet based on maize, and eggs were removed daily. All hens were in the laying period and a balanced selection of hens between the ages of 47 and 67 weeks was made. The animals were euthanized by CO_2_ anesthesia and decapitated with scissors. After exsanguinating the animals, the abdomen was opened and the ceca were removed on the *basis ceci* and immediately transferred to the lab in preheated and carbogen-gassed Krebs-Henseleit-buffer (KH).

### 2.2. Buffers and Solutions

For the transport and preparation of the smooth muscle strips and for the incubation in the organ bath, KH was used (in mmol/L: NaCl 115.0 (VWR International GmbH, Darmstadt, Germany), 4.7 KCl, 2.5 CaCl_2_, 1.2 MgCl_2_, 1.2 NaH_2_PO_4_, 25.0 NaHCO_3_ (all Carl Roth GmbH & Co. KG, Karlsruhe, Germany) and 11.0 glucose (VWR International GmbH, Darmstadt, Germany)). Before use, the buffer was gassed with carbogen (95% O_2_, 5% CO_2_) under constant temperature conditions (38 °C). The final pH of the buffer was 7.4. Furthermore, two buffer variations were used for the experiments: a calcium-free buffer (in mmol/L: NaCl 115.0 (VWR International GmbH, Darmstadt, Germany), 4.7 KCl, 1.2 MgCl_2_, 1.2 NaH_2_PO_4_, 25.0 NaHCO_3_ (all Carl Roth GmbH & Co. KG, Karlsruhe, Germany) and 11.0 glucose (VWR International GmbH, Darmstadt, Germany)) and a potassium-enriched buffer, containing potassium chloride instead of sodium chloride (in mmol/L: 123.4 KCl, 2.5 CaCl_2_, 1.2 MgCl_2_, 1.2 NaH_2_PO_4_, 25.0 NaHCO_3_ (all Carl Roth GmbH & Co. KG, Karlsruhe, Germany) and 11.0 glucose (VWR International GmbH, Darmstadt, Germany)).

### 2.3. Tissue Preparation

Apex ceca were removed from the intestine, cecal content was removed and the tissue was washed using prewarmed KH. Ceca were stored for a maximum of 20 h in pre-gassed (95% O_2_, 5% CO_2_) KH at 4 °C until further processing. Preparation of the smooth muscle layer was made by opening the ceca along the mesenterial side and carefully removing the mucosa and submucosa with forceps and scissors under a binocular light microscope (Carl Zeiss AG, Oberkochen, Germany). Then, muscle strips were cut parallel to the circular smooth muscle fibers, of about 2 mm width and 15 mm length, with a scalpel. A grid was used to ensure that the muscle strips were mostly equal in size and weight. Each muscle strip was then mounted in an organ bath filled with 5 mL KH and fixed on an isometric force transducer (complete equipment from Hugo Sachs Elektronik—Harvard Apparatus GmbH, March, Germany). Initial tension was adjusted at 20 mN, which was tested in previous studies to be the optimal pretension to obtain maximal muscle contraction, and the muscle strip was equilibrated for 30–60 min. Measurement started when spontaneous muscle contractions occurred frequently for at least 2 min. One animal per day was used for the experiments using four organ baths in parallel, measuring four to eight muscle strips per animal. After the experiment, the muscle strips were dried at 60 °C for six days to determine their dry weight.

### 2.4. Isometric Force Measurement of the Muscle Contraction

After the equilibration time, the spontaneous contraction of the smooth muscle was measured for two minutes. Frequency, mean peak height and the intensity of the peak as the area under the curve (AUC) were detected. Muscle strips not showing spontaneous contraction were discarded and replaced. Muscle contraction was then stimulated using different buffers and solutions. First, KH was replaced by calcium-free buffer or potassium-enriched buffer. Carbachol (VWR International GmbH, Darmstadt, Germany; final concentration: 10^−9^ mol/L), nitroprusside (Alfa Aesar, Ward Hill, MA, USA; final concentration: 2.5 × 10^−3^ mol/L), isoprenaline (Acros organics, Fair Lawn, NJ, USA; final concentration: 10^−6^ mol/L) and Verapamil (VWR International GmbH, Darmstadt, Germany; final concentration: 2 × 10^−6^ mol/L) were added to the organ bath in subsequent steps. Furthermore, atropine was used (final concentration: 10^−6^ mol/L) 5 min before carbachol was added. The tissue was washed with KH between the different treatments and the organ bath was refilled with fresh KH. Except with Verapamil, all effects were reversible when the substances were washed out. Treatments of the individual muscle strips were chosen depending on the vitality of the muscle tissue. Muscle strips without stable physiological contraction were discarded. This led to a subset of substances and buffers with the minimum numbers of replicates defined as n ≥ 6 and N ≥ 4 (Table S1), respectively. The influence on the muscle contraction was recorded for two minutes after each treatment. 

### 2.5. Data Analyses

The parameters were recorded and analyzed by ACAD software (Hugo Sachs Elektronik, Harvard Apparatus GmbH, March, Germany). Contractions were evaluated based on the height, duration and frequency of the peaks. The results are shown as frequency (contractions/2 min), peak height as isometric force (mN) and total isometric force (mN × 2 min^−1^), which represents the peak intensity measured as the area under the curve. The data refer to an observation time of 2 min for each treatment. As recommended by the company, a deflection of more than 3 mN above the basal line was defined as a peak assuming that it was above an amplitude caused by vibrations in the lab building. The baseline was calculated by the average of the basal values. Measurements started directly after the substance was added. The comparability of the results was determined by referring all values to a muscle weight of 1 mg and giving them as means ± SEM with N = number of animals. The significance was calculated by JMP Pro 15 software (SAS Institute, Cary, NC, USA) using a Wilcoxon test and defined as *p* = 0.05. 

## 3. Results

### 3.1. Weight of the Muscle Strips

The standardized preparation of the muscle strips yielded a mean dry weight of 1.2 ± 0.1 mg of each muscle strip.

### 3.2. Spontaneous Contraction

**Figure 1:**
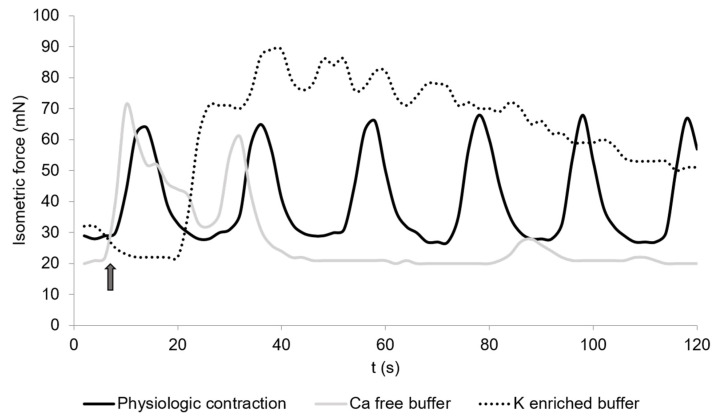
Spontaneous contractions of chicken circular cecal smooth muscle. The control condition is given (physiological contraction, black) and contractions during the incubation in calcium-free buffer (dotted) or potassium-enriched buffer (light grey). The arrow shows the time when the buffers were changed. The contraction was detected for 2 min. The graph is a typical example from a total of 36 measurements.

**Figure 2:**
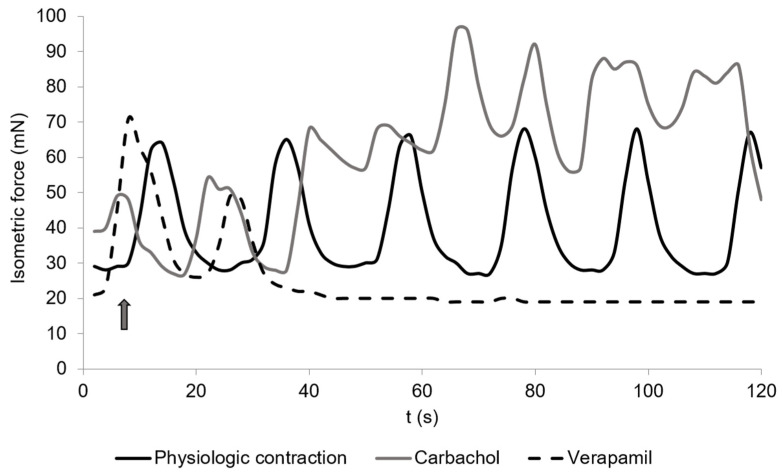
Spontaneous contractions of chicken circular cecal smooth muscle. The control is given (physiological contraction, black) and the reaction to carbachol (10^−9^ mol/L, dark grey) or Verapamil (2 × 10^−6^ mol/L, dashed). The arrow shows the time when drugs were added. The contraction was detected for 2 min. The graph is a typical example from a total of 36 measurements.

**Figure 3:**
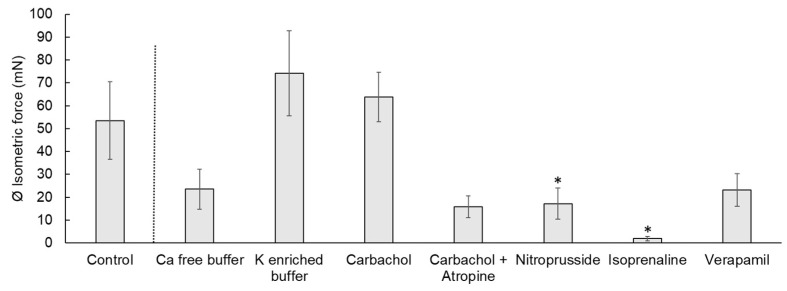
Isometric force of the contraction of chicken circular cecal smooth muscle. The control condition and the reactions to different electrolytes or drugs are shown. Values are given as mean ± standard error of the mean (SEM). The treatments were compared to the control condition and significant differences are indicated by * at *p* < 0.05.

**Figure 4:**
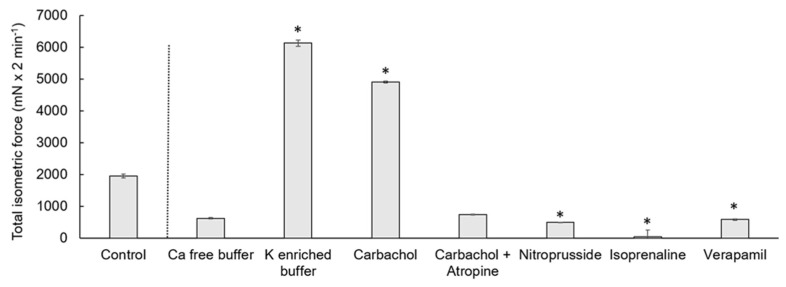
Total isometric force measured as the AUC of cecal circular smooth muscle contraction. The control condition and the reactions to different electrolytes or drugs are shown. The values are given as mean and standard error of the mean (SEM). The treatments were compared to the control condition and significant differences are indicated by * at *p* < 0.05.

The contraction measures of the cecal smooth muscle strips are exemplified in Figure 1 and Figure 2. Spontaneous contractions of the muscle strips had a mean isometric force of 53.5 ± 16.9 mN (Figure 3). The mean peak frequency was 6.2 ± 0.7 in two minutes (Table 1) with a total isometric force of 1962.9 ± 604.3 mN × 2 min^−1^ (Figure 4). The data of the individual animals are shown in Table S1.

### 3.3. Calcium-Free Buffer

Calcium-free buffer resulted in a significant decrease (*p* = 0.001) of the peak frequency (1.3 ± 0.3) (Figure 1, Table 1). The mean isometric force was 23.5 ± 8.7 mN and the total isometric force was 632.8 ± 268.2 mN × 2 min^−1^, which was significantly lower compared to the control (Figure 3 and Figure 4). Complete elimination of the contraction activity was reached after a mean of 29.7 ± 6.8 s.

### 3.4. Potassium-Enriched Buffer

Potassium-enriched buffer significantly increased (*p* = 0.0334) the total isometric force (6131.9 ± 2093.8 mN × 2 min^−1^) (Figure 1 and Figure 4), but induced a significant decrease (*p* = 0.0004) in frequency (1.5 ± 0.2) (Table 1) compared to the control condition. The mean isometric force was 74.2 ± 18.5 mN (Figure 3).

### 3.5. Carbachol

The addition of carbachol to the KH buffer resulted in an isometric force of 63.7 ± 10.8 mN (Figure 2 and Figure 3) and a significant increase of the total isometric force (4907 ± 1006.9 mN × 2 min^−1^) (Figure 4). The peak frequency was 2.2 ± 0.9 (Table 1). It must be noted that the applied concentration (10^−9^ mol/L) was evaluated carefully as higher concentrations of carbachol resulted in strong contractions, which were out of the measurement ranges of the used equipment.

### 3.6. Carbachol and Atropine

Atropine was used to inhibit the carbachol reaction as it acts as an anticholinergic drug [27] that binds to the muscarinic receptors. No response to carbachol was observed when the tissue was pre-incubated with atropine for 5 min.

### 3.7. Nitroprusside

Nitroprusside significantly (*p* ≤ 0.05) lowered the mean (17.2 mN ± 6.8 mN) (Figure 2 and Figure 3) and the total isometric force (507.6 ± 192.9 mN × 2 min^−1^) (Figure 4) compared to the control condition. The frequency (3.5 ± 0.8) was also significantly reduced (*p* = 0.0349) (Table 1).

### 3.8. Isoprenaline

Isoprenaline caused a significant (*p* ≤ 0.05) depression of the muscle contraction, resulting in a mean isometric force of 1.9 ± 1.0 mN, frequency of 0.8 ± 0.4 and total isometric force of 48.2 ± 13.9 mN × 2 min^−1^ (Figure 3 and Figure 4, Table 1).

### 3.9. Verapamil

Verapamil significantly (*p* = 0.0185) inhibited the total isometric force (592.0 ± 199.4 mN × 2 min^−1^) (Figure 2 and Figure 4), but also seemed to inhibit (*p* = 0.0794) the frequency (3.9 ± 0.8) (Table 1). The mean isometric force was 23.2 ± 7.2 mN (Figure 3).

## 4. Discussion

The current study investigated cecal smooth muscle contraction in physiological conditions, detecting spontaneous muscle contraction. Further to that, the contraction was stimulated by different buffers and solutions. Thereby, calcium-free buffer and Verapamil were used to test the calcium dependency of the contraction. Potassium-enriched buffer was applied to induce hyperpolarization of the cell membrane and to investigate the effect on muscle contraction. The influence of the sympathetic and parasympathetic nervous systems was tested using carbachol, atropine and isoprenaline. Nitroprusside was used to study the influence of the neurotransmitter nitric oxide (NO).

The mean isometric force, total isometric force and frequencies of the muscle tissue in physiological conditions and during the treatments were measured. These parameters are important indicators of intestinal motility, as this depends on the interaction of individual muscle fibers. The contraction and relaxation of the muscle fibers influence the mixture and transport of the digesta and thus stimulate the digestion of nutrients. The mean isometric force is a parameter for the contraction force of the muscle fibers. A higher contraction force could enable better mixture and faster transport of the digesta. The duration of a contraction is considered by the total isometric force, which is important for the transport velocity of the digesta. The frequency evidences the abundance of contractions in a defined period. Depending on the direction of the contraction, a high frequency can imply an improved mixture of the digesta or faster transport. Nevertheless, the muscle fibers are working as a syncytium and the parameters described above should always be analyzed as a whole as they also affect each other. Comparison of the present measurements to literature data is difficult as there is a lack of data about cecal smooth muscle contraction in chickens. Therefore, most of the presented data will be compared to similar studies in other species. As different species differ in their nutrition and gastrointestinal physiology, a true comparison is not possible.

An in vitro study using whole cecal segments from broilers showed an average frequency of 14 contractions (physiological conditions) per minute using longitudinal smooth muscles instead of circular smooth muscles [18]. A true comparison to the present data is not possible as circular and longitudinal smooth muscles differ in their contraction and behavior, and larger amounts of muscle cells generate different measures. Thus, more research is needed to compare and evaluate the results and to provide more information about cecal contraction and motility. In the jejunum of horses, a frequency of five contractions/min was observed with a mean isometric force of 25 mN [26]. In the small intestine of dogs, a maximal frequency of 14 contractions/min in the ileum and 18 contractions/min in the duodenum was detected [28]. These data showed that the contraction frequency differs between different species and intestinal sections, and a frequency of six contractions in two minutes in the ceca of laying hens seems to be realistic.

The replacement of KH by calcium-free buffer led to a depression of the total isometric force and frequency in the muscle strips. Calcium plays a major role in the activation of smooth muscle contraction. Without calcium, cross-bridge cycling cannot take place, resulting in declining and an end to muscle contraction. Studies in mammals and bufos confirmed the absence of spontaneous smooth muscle contractions during incubation in calcium-free solution [29].

In contrast, potassium-enriched buffer increased the total isometric force but also induced a significant decrease in frequency. An increased concentration of extracellular potassium causes continuous depolarization of the electrical potential across the cell membrane. This results in a lasting opening of the potential-dependent Ca^2+^ channels and cell contracture, which is defined by an initially decreased frequency of contractions and complete absence of contractions at later stages. The same effect was shown before in circular and longitudinal muscle strips of the small intestine [30] and in the gastric smooth muscle of dogs [31,32].

Carbachol is a parasympathomimetic drug, which binds on the muscarinic acetylcholine receptors M_2_ and M_3_ of the muscle cells, and consequently leads to contraction of the smooth muscles of the gastrointestinal tract [33,34]. Thereby, stimulation of M_3_ leads to an initial contraction of the smooth muscle cells, as it stimulates the mobilization of Ca^2+^. The activation of M_2_ does not directly lead to muscle contraction but has a potentiating effect on the M_3_ receptor and other receptors mediating the release of Ca^2+^ by initiating a parallel signaling pathway [34,35]. Atropine acts as an anticholinergic drug [27] that binds to the M_2_ and M_3_ receptors and blocks the carbachol reaction, as was shown in the present experiments.

Nitroprusside lowered the isometric force and frequency compared to control conditions. Nitroprusside is an NO donator inducing relaxation of the smooth muscle. NO is a non-adrenergic and non-cholinergic neurotransmitter that increases the cyclic guanosine monophosphate (cGMP) level, especially in the intestinal smooth muscles [12,36]. cGMP activates an intracellular molecular cascade, resulting in a depletion of free Ca^2+^ in the cytosol, which leads to an inhibition of smooth muscle contraction. Thereby several mechanisms are involved: the activation of protein kinase G (PKG) causing phosphorylation of the proteins that are responsible for the intracellular Ca^2+^ concentration, such as ion channels and ion pumps; the activation of K^+^ channels causing hyperpolarization of the cell membrane and thus inhibition of the Ca^2+^ channels and activation of the Ca^2+^/ATPase pump in the plasma membrane and sarcoplasmic reticulum. Furthermore, cGMP leads to reduced Ca^2+^ sensitivity [37]. However, different studies of extra-intestinal smooth muscles indicated that part of the NO-induced smooth muscle relaxation is independent of cGMP [38,39,40,41,42].

Isoprenaline caused a significant depression of muscle contraction. This belongs to the catecholamines and acts as a β-sympathomimetic drug [43]. The effect of catecholamines on intestinal smooth muscle contraction was shown to be caused by two mechanisms. First, the presynaptic release of acetylcholine in the intramural plexuses was inhibited [44,45], which consequently led to downregulation of smooth muscle contraction. Second, it is assumed that catecholamines interact directly on smooth muscle cells via α- and β-adrenoceptors [43,46]. Both mechanisms cause relaxation of the smooth muscle, associated with decreases in frequency, amplitude and peak intensity. The same reaction can be induced by the sympathetic nervous system. The effect was already shown in other animals, including guinea pigs and dogs [45,47].

Verapamil resulted in a decline up to a complete stop in muscle contraction as it blocks the calcium channels in the smooth muscle cell membranes [48].

In the current study, spontaneous contraction of the chicken circular cecal smooth muscle was recorded. Furthermore, the influence was tested of electrolytes and drugs that stimulate smooth muscle contraction via different physiological pathways, such as the cross-bridge-cycling mechanism, the membrane potential and the sympathetic and parasympathetic influence. High inter-individual variation regarding smooth muscle activity has to be considered, as indicated by the standard error of the mean, even if hens are kept and fed under the same conditions. Nevertheless, the results of the present study provide the first key characterization of the contraction of cecal smooth muscle in chickens. 

## 5. Conclusions

Smooth muscle contraction is important for gut motility and the overall digestive process. In this study, the contraction of the cecal smooth muscle was studied and stimulated by the addition of different electrolytes and drugs. The general effects were comparable to studies in other animals. Confirmation of the present results and comparison with other studies in chickens’ cecal smooth muscles was not possible as almost no data are available. Hence, more research is required to verify the present findings. Furthermore, the relevance of the responses to the applied electrolytes and drugs on intestinal motility and digestion in hens is still unclear. However, the current data represent the first important findings and can be used as a basis for further research regarding the physiology of chickens’ digestive tract. This could also offer the chance to investigate new medical options for intestinal dysfunctions in the birds as modern animal welfare and protection strategies strive toward sustainable animal production.

## Figures and Tables

**Table 1:** Frequency (contractions/2 min) of the physiological smooth muscle contraction of the cecum and changes in the frequency as affected by electrolytes and drugs. The results show frequencies for the control conditions (physiological contraction) and treatments. The values are given as mean and standard error of the mean (SEM). Significant differences were defined as *p* < 0.05 between the treatments and the physiological contractions. n.s. = not significant. -= not defined. N = number of animals.

	Control	Ca-Free Buffer	K-Enriched Buffer	Carbachol	Carbachol +Atropine	Nitroprusside	Isoprenaline	Verapamil
Frequency	6.2	1.3	1.5	2.2	5.6	3.5	0.8	3.9
SEM	0.7	0.3	0.2	0.9	1.1	0.8	0.4	0.8
*p*–Value	-	0.0010	0.0004	0.0049	n.s.	0.0349	0.0004	n.s.
N	11	7	9	8	4	11	8	9

## Data Availability

The datasets generated and/or analyzed during the current study are available from the corresponding author on reasonable request.

## Supplementary Materials

The following are available online at https://www.mdpi.com/article/10.3390/vetsci8060091/s1: Table S1, All measurement data obtained through the experiments.
